# Chronic pramipexole treatment increases tolerance for sucrose in normal and ventral tegmental lesioned rats

**DOI:** 10.3389/fnins.2014.00437

**Published:** 2015-01-06

**Authors:** David Dardou, Carine Chassain, Franck Durif

**Affiliations:** ^1^EA7280 NPSY-Sydo, Université d'AuvergneClermont-Ferrand, France; ^2^IRM-Hopital Gabriel MontpiedClermont-Ferrand, France; ^3^Service de Neurologie A, Hopital Gabriel MontpiedClermont-Ferrand, France

**Keywords:** sucrose tolerance, ventral tegmenta area, Parkinson's disease, dopamine agonist, pramipexole, food compulsion

## Abstract

The loss of dopamine neurons observed in Parkinson's disease (PD) elicits severe motor control deficits which are reduced by the use of dopamine agonists. However, recent works have indicated that D3-preferential agonists such as pramipexole can induce impulse control disorders (ICDs) such as food craving or compulsive eating. In the present study, we performed an intermittent daily feeding experiment to assess the effect of chronic treatment by pramipexole and VTA bilateral lesion on tolerance for sucrose solution. The impact of such chronic treatment on spontaneous locomotion and spatial memory was also examined. Changes in sucrose tolerance could indicate the potential development of a change in food compulsion or addiction related to the action of pramipexole. Neither the bilateral lesion of the VTA nor chronic treatment with pramipexole altered the spontaneous locomotion or spatial memory in rats. Rats without pramipexole treatment quickly developed a stable intake of sucrose solution in the 12 h access phase. On the contrary, when under daily pramipexole treatment, rats developed a stronger and ongoing escalation of their sucrose solution intakes. In addition, we noted that the change in sucrose consumption was sustained by an increase of the expression of the Dopamine D3 receptor in the core and the shell regions of the nucleus accumbens. The present results may suggest that long-term stimulation of the Dopamine D3 receptor in animals induces a strong increase in sucrose consumption, indicating an effect of this receptor on certain pathological aspects of food eating.

## Introduction

The loss of dopamine (DA) producing neurons observed in Parkinson's disease (PD) elicits severe motor control deficits including rest tremor, bradykinesia, and rigidity. While dopamine replacement therapy (DRT) reduces motor symptoms, a growing number of studies have shown that the use of DRT, in particular dopamine agonists, induces impulse control disorder (ICD) such as dopamine dysregulation syndrome, hypersexuality, pathological gambling, compulsive shopping, and disturbance of eating behavior (Courty et al., 1997; Gallagher et al., 2007; Voon and Fox, 2007; Merims and Giladi, 2008; Weintraub et al., 2010). Clinical studies have shown that PD patients occasionally develop food craving or compulsive eating disorder (Nirenberg and Waters, 2006; Miwa and Kondo, 2008; Rieu et al., 2011). Such abnormal behavior generally induces a significant, undesired weight gain that can disturb the PD patient's quality of life.

Progressive loss of dopaminergic neurons leads to the denervation of both the nigro-striatal pathway (around 80%) from the substantia nigra (SN) and the mesocorticolimbic system (around 40%) from the ventral tegmental area (VTA) (German et al., 1989). Although the loss of dopaminergic neurons in the SN is directly linked to the motor and apathy symptoms observed in PD patients and animal models (Dagher and Robbins, 2009; Drui et al., 2013), the relation between the lesion of the dopaminergic brain region and ICD remains unclear. Although the lesion of the VTA in Parkinsonian patients is rather limited, the loss of dopaminergic neurons in this brain region could account, at least partially, for the development of ICD in PD patients under DRT. Indeed, the main dopaminergic afferent projections coming from the VTA are directed to the nucleus accumbens (Acb). The dopamine arising from these afferent projections acts on the Dopamine D2 and D3 receptors in the Acb. This nucleus plays a key role in the regulation of the mesocorticolimbic system (Robinson and Berridge, 2001) due to its place in the brain's reward circuit.

Dopamine agonists, particularly pramipexole (PPX), induces conditioned place preference in normal animals as well as those with 6-OHDA lesions of the striatum or SN (Papp, 1988; Riddle et al., 2012; Zengin-Toktas et al., 2013). Interestingly, it was observed that sham-operated animals need a higher dose of PPX to induce conditioned place preference compared to rats with dopaminergic lesions in the striatum, pointing to potentially higher sensitivity to the reward properties of PPX in rats with lesions (Riddle et al., 2012). In addition, recent works have found that rats with lesions of the posterior region of the VTA develop a conditioned place preference induced by D2 and D3 agonists (Ouachikh et al., 2013), while lesion of the anterior region of the VTA disrupts the ability of rats to learn conditioned place preference (Ouachikh et al., 2014). Although the conditioned place preference paradigm appears to be limited to the study of motivational aspects linked to ICD, such data could indicate possible interaction between dopamine agonist treatment and lesion of the VTA region, potentially leading to a change in motivated behaviors. In addition, recent clinical studies from Thobois et al. (2010, 2013) have shown that non-motor symptoms (apathy, depression, and anxiety) was more expressed in patients with enhanced denervation of mesocorticolimbic pathway.

If VTA denervation contributes to CPP learning in rodents and to apathy, depression, and anxiety in PD patients, it is possible that it could also participate in certain aspects of impulsive/compulsive disorders. To address this question, we chose in the present work to investigate the effect of VTA lesion and dopamine agonist treatment in a model of compulsive food disorder, i.e., intermittent daily feeding. Intermittent daily feeding is a well-described behavioral animal model of food addiction or binge eating in which rats develop tolerance to sucrose solution by successive day-to-day access as well as signs of withdrawal when access to sucrose is removed (Avena et al., 2008a,b; Avena, 2010). Using this behavioral approach, we investigated the effect of the lesion of the VTA region and chronic PPX administration on sucrose solution tolerance, which preferentially activates the D3 receptor at low doses (Collins et al., 2005). Interestingly, an increase of D3 mRNA expression in the neurons of the direct striatonigral pathway was observed in rats with 6-OHDA lesion under chronic administration of L-Dopa (Bordet et al., 2000). Furthermore, this receptor is implicated in certain aspects of drug dependence such as drug seeking and relapse (Le Foll et al., 2005). Blockade of the D3 receptor by a selective antagonist has been reported to transiently alleviate craving to nicotine in smoking addicts (Mugani et al., 2013) and to disrupt morphine triggered cocaine induced CPP in rats (Rice et al., 2013).

The aim of the present study was to assess the effect of pramipexole and bilateral VTA lesions in rats on tolerance for sucrose solution using a model of daily intermittent sucrose access, which is considered to mimic aspects of compulsive food eating in humans. In addition, we also explored the effect of such chronic treatment on motor, cognitive, and anxiety-like traits. In addition, change in D3 expression in the Acb was explored.

## Materials and methods

### Subjects

Forty-five male Sprague Dawley rats (Charles River, Les Oncins, France) weighing 200–225 g were housed individually. Animals had access *ad libitum* to food and water before the behavioral studies were started. All the experiments were carried out in conformity with the European Committee Council Directive of 24 November 1986 (86/609/EEC) and were approved by the local ethical committee (CEMAAuvergne).

### Bilateral 6-OHDA lesion and pramipexole treatment

Animals were anesthetized by an i.p. injection of mixture of ketamine (60 mg/Kg) and xylazine (20 mg/Kg) and treated with desipramine (25 mg/Kg, i.p., Sigma France) 30 min before 6-OHDA injection. Bilateral lesions of the VTA were performed by micro injection of 6-OHDA (2 μg/μL, dissolved in NaCl 0.9% with 0.01% of ascorbic acid solution, Sigma France) at the following coordinate: AP: –5.2 mm; Lat: ± 0.8 mm; DV: –7.6 mm from the bregma at a speed of 0.125 μL/min for a total of 1 μl injected on each side. The syringe was left in position for at least 10 min to reduce diffusion along the needle tract. Sham-operated animals received the same procedure with the infusion of the vehicle (NaCl 0.9% with 0.01% of ascorbic acid solution, Sigma France). Animals had a 2-week rest period to regain normal home caged behaviors and to stabilize the 6-OHDA lesion.

**Figure 1:**
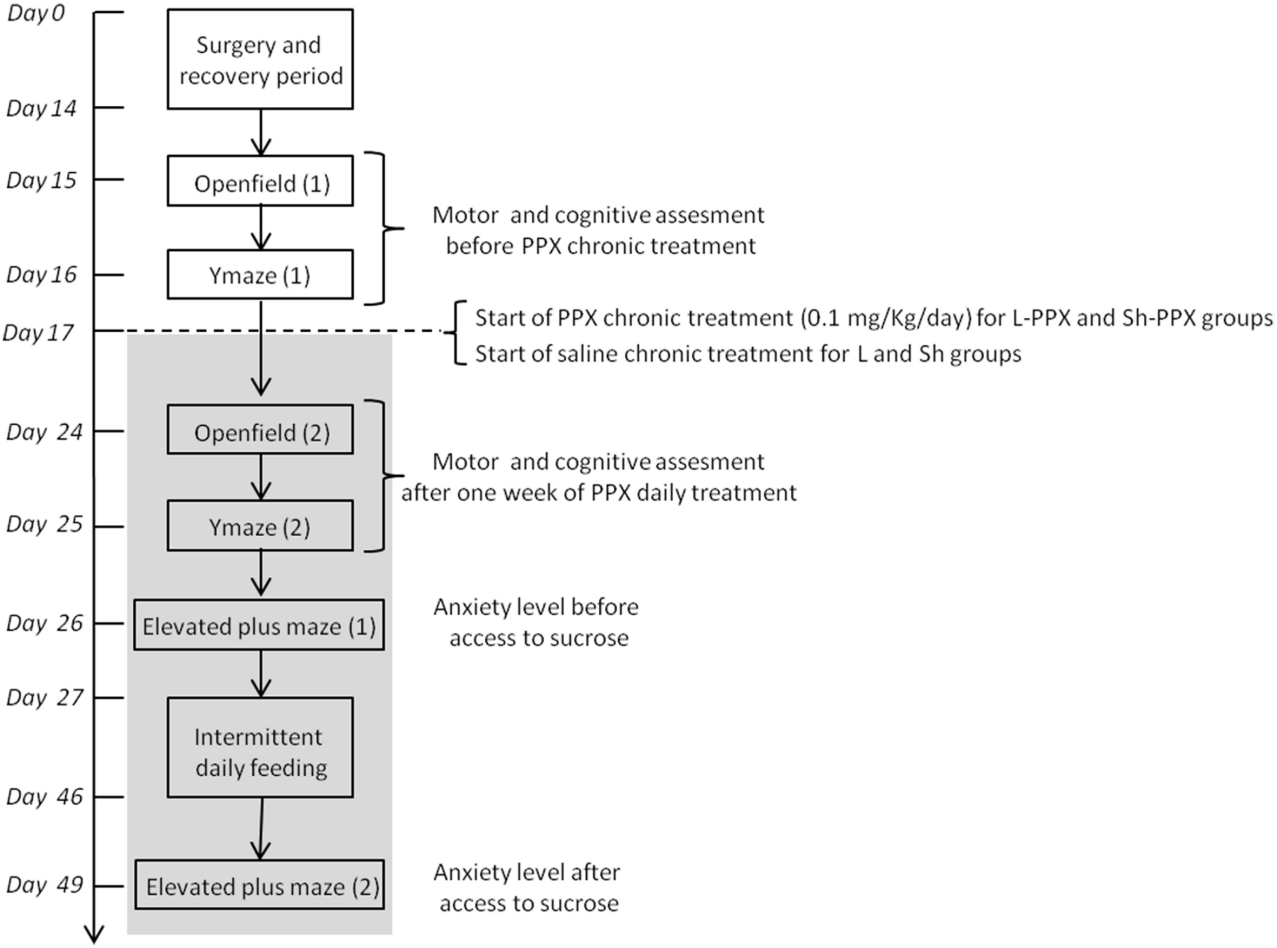
**Time schedule of behavioral experiments**. After a period of acclimation to animal house, rats received 6-OHDA or vehicle solution injection in the VTA. After the recovery period of 2 weeks, all animals were evaluated for spontaneous locomotion and spontaneous alternation in Y maze on 2 days. After the first evaluation, chronic PPX treatment starts with daily administration of 0.1 mg/kg/day of PPX by s.c. injection and last until the end of the experiment (light gray box). A second evaluation of spontaneous locomotion and spontaneous alternation in Y maze was performed after 1 week of PPX treatment. The anxiety level of animal was measured using elevated plus maze, the day before and 2 days after the last day of the 10% sucrose solution access. Intermittent daily feeding was assessed during 19 consecutive days.

Two groups of rats (sham and 6-OHDA lesioned rats) were treated daily with an s.c. injection of pramipexole (PPX, Sigma France) at a dose of 0.1 mg/kg/day (dissolved in 0.9% NaCl). PPX was administered every morning at 7 a.m. by subcutaneous injection from the 17th experimental day (Figure 1) to the end of the experiment (day 49, so rats were treated for 32 days). *Two* other control groups (sham and 6-OHDA lesioned rats) were s.c. injected daily with 0.9% NaCl solution. Lesioned and sham animals treated with PPX are referred to as L-PPX (*n* = 12) and Sh-PPX (*n* = 10), respectively. The control groups are referred to as L (*n* = 12) and Sh (*n* = 11) for the lesioned and sham groups, respectively. The treated animals received an injection once a day at the same time (7 a.m.) and at least 2 h before behavioral tasks took place.

### Behavioral testing

After a recovery period, all the animals completed the following sequence of behavioral tests (Figure 1). Animals were evaluated in open field box and alternation in a Y maze in order to estimate spontaneous locomotor activity and spatial memory before and after 1 week of PPX treatment, respectively. The anxiety level of animals was measured the day before and 2 days after the last day of access to the 10% sucrose solution.

#### Y maze

Alternation in the Y maze made of gray plastic was performed under soft light (30–35 lux). It consisted of three identical branches (45 cm long, 13 cm wide, and 20 cm high) which diverged at an angle of 120° from the central point. Animals were placed in the start branch and freely explored the maze for 5 min. The orders of the visits were noted by the observer. The maze was cleaned with hot water and ethanol between each run. Alternation was determined from successive entries (four paws inside the branch) of the three branches by overlapping triplet sets in which three different branches were entered (Taghzouti et al., 1986; Pioli et al., 2008). The alternation score was obtained by dividing the number of alternations by the number of alternation opportunities (the total branch entries minus 2).

#### Open field

Spontaneous motor activity was measured in an open field box (75 cm long, 75 cm wide, and 45 cm high) placed under a camera used to record each trial under soft lighting (30–35 lux). Animals were placed in the center of the box and were allowed to move freely during 15 min. All sessions were video-recorded and analyzed with EthoVision XT 8.5 (Noldus, Netherlands). The open field arena was then cut in 16 squares of identical size for analysis. The mean distance run (in cm) and number of squares crossed and the numbers of rearings were analyzed.

#### Elevated plus maze

Anxiety behavior was assessed using an elevated plus maze (50 cm long, 10 cm wide, 10 cm high for the protected arms, 52 cm from the ground) under soft illumination (30–35 lux) and a camera was used to record all the trials. Rats were placed in the center of the device and were free to move for 5 min. The time spent in open arm ratio (OTR) was calculated by dividing the times spent in the open arm by the time spent in the open and closed arms. OTR was used as the measure of anxiety as well as the number of entries in the non-protected arms in comparison to the protected one. The experiment was analyzed using EthoVision XT 8.5 (Noldus, Netherlands).

#### Intermittent daily feeding

All experimental groups were placed under daily alternation for 19 days. During this period, animals received a drinking bottle containing a 10% sucrose solution at the start of the dark phase (12 h). The present protocol was adapted from the studies of Avena et al. (2008a,b) to our animal house condition. The bottles containing the 10% sucrose solution was given to rats immediately at the start of the dark period (7 p.m.) and removed at the start of the light phase (7 a.m.). Animals had access to tap water only during the light phase. The amount of sucrose solution (in g.) was noted by weighing the bottle before and after the dark period. Food was accessible *ad libitum* throughout the experiment.

#### Detection of tyrosine hydroxylase and dopamine D3 receptor expression

After the completion of the behavioral evaluations, rats were killed by chloral hydrate injection. The brains of the animals were collected and post-fixed for 24 h in PFA 4%, before being processed for paraffin embedded sections. Paraffin blocs were then serially cut into 7 μm thick sections (first slice chosen randomly) and mounted on glass slides.

Detection of tyrosine hydroxylase (TH) was used to determine the extent of the 6-OHDA lesion. Paraffin was removed by successive baths of xylene, ethanol and bathing in hot citrate buffer for 25 min. After cooling at room temperature, the slides were rinsed in tris-phosphate buffer 0.1 M (TBS 0.1 M) and endogeneous peroxydases were quenched with TBS 0.1 M with 5% H_2_O_2_ for 30 min. Sections were then incubated with anti-TH antibody (1/5000, rabbit, AB152 Milipore USA) diluted in DAKO REAL antibody diluents overnight at room temperature. The next day, the presence of the epitope was revealed using the DAKO LSAB and HRP system (DAKO Real, DAKO France). The reaction allowed localizing the expression of TH by brown labeling on the slides. In order to reduce the differences in background labeling between all the slides, all the slides from the experiments were processed at the same time for TH immunohistochemistry under the same laboratory conditions. TH-positive neurons were counted using ImageJ software on 12 slices from the anterior to posterior axis of the brain. The numbers of positive cells in both VTA and SN were estimated with the method described by Paillé et al. (2007).

Slices containing the accumbens and striatum at the level around +2.16 mm from Bregma were processed for the detection of Dopamine D3 receptor expression. After pretreatment, the slices were incubated at room temperature with TBS solution containing 3% normal goat serum (NGS, Jackson ImmunoResearch USA) for 1 h. After rinsing, the slices were incubated overnight at 4°C with a solution of TBS containing 1% NGS and anti-dopamine D3 receptor antibody (1/200, rabbit polyclonal, sc-9114, Santa Cruz USA). The next day, the slides were rinsed using TBS and incubated with secondary antibody (goat anti rabbit alexa 488, Jackson ImmunoResearch USA) for 1 h at room temperature. The slides were rinsed and mounted using Dako fluorescent mounting medium and kept in the dark at 4°C before analysis. In order to reduce the differences in background labeling between all the slides, all the slides from the experiments were processed at the same time for D3R immunohistochemistry under the same laboratory conditions. Observation of the fluorescent labeling was done using a Zeiss microscope and AxioVision software. The number of positive cells was counted in 100 μm^2^ square windows, positioned at the same place using a brain atlas. In the Acb, one window was used for the core region and one window for the shell region. The numbers of positive cells was noted as the mean number of cells per 100 μm^2^.

### Data analysis

All behavioral data were expressed as mean ± S.E.M. for each experimental group and analyzed using a Two-Way repeated-measures analysis of variance (ANOVA) with lesion (two levels, lesioned and sham) and treatment (two levels, PPX or saline) as within subject factors. When appropriate, data were compared using the Tukey–Kramer *post-hoc* test for multiple comparisons.

For sucrose consumption, the intake of sucrose was also expressed using the area under curves (A.U.C.). The A.U.C. was obtained by summing the squares under the curves for each group using the trapezoidal rule as followed: Σ [(VTtime X – VTtime 0) (time X – time X-1)]. The A.U.C. values, the mean number of TH positive cells and the mean number of D3 positive cells were analyzed by a One-Way ANOVA with the experimental groups (four levels, L or L-PPX or Sh or Sh-PPX) as the factor and, when appropriate, data were compared using Tukey–Kramer *post-hoc* tests for multiple comparisons. Statistical analysis was performed with SAS 9.1 software (SAS institute Inc., USA).

## Results

### Bilateral partial lesions of the VTA

**Figure 2:**
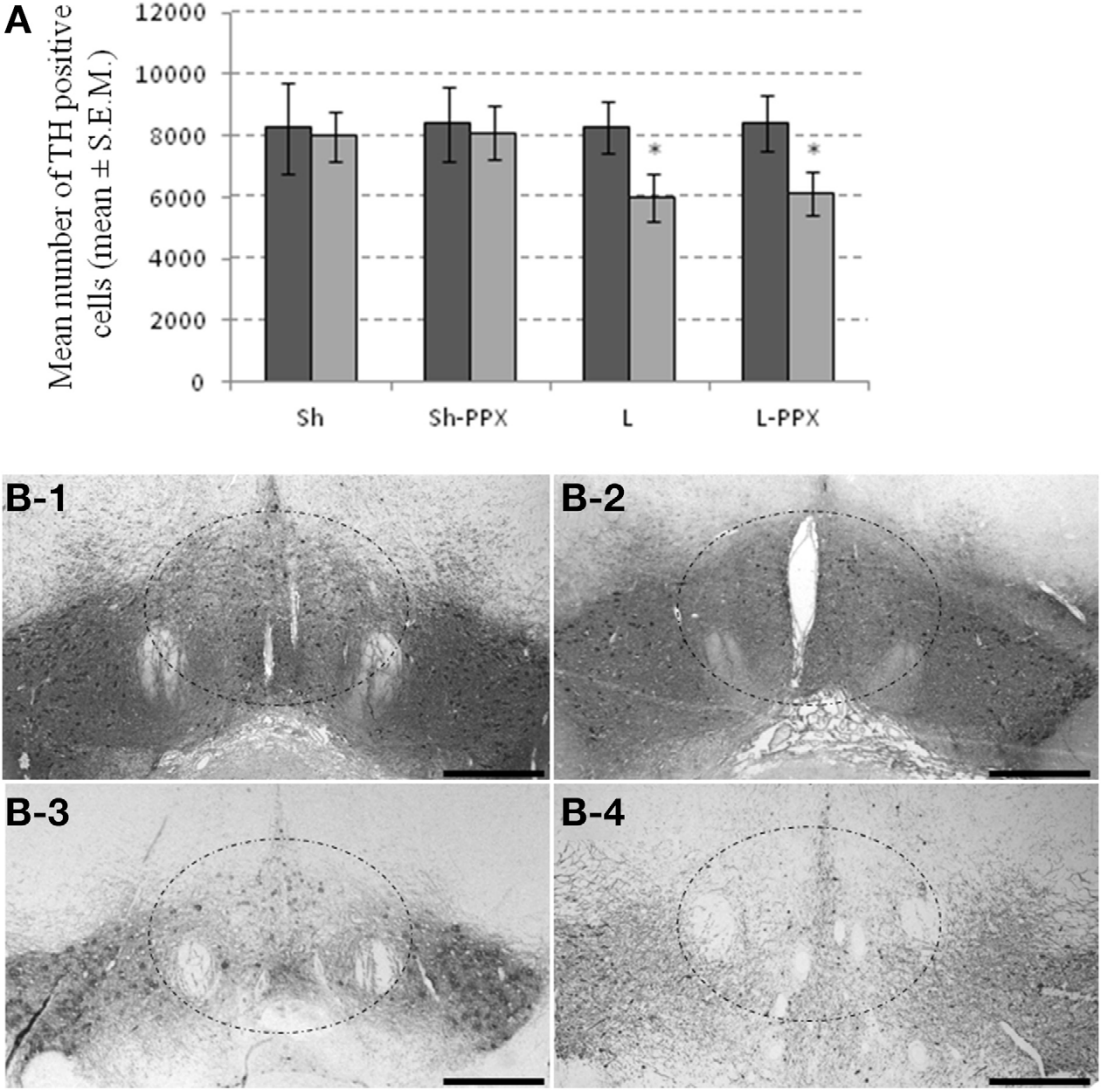
**Partial bilateral lesion of VTA by 6-OHDA micro-injection**. The injection of 6-OHDA produced a decrease in the number of TH-positive cells within the VTA of lesioned rats **(A)**. The dark gray bar represented the number of positive cells counted in the SN, while light gray bar represented the number of positive cells counted in the VTA region. Microphotographs illustrated the dopamine denervation following 6-OHDA injection in Sh **(B-1)**, Sh-PPX **(B-2)**, L **(B-3)**, and L-PPX **(B-4)**. The dash line square represents the region where lesion is the most representative. Black scales bars represent 100 μm. Microphotographs were taken around −5.28 mm from the Bregma.

The effect of the bilateral micro-injection of 6-OHDA revealed by TH-immunoreactivity indicated a discrete pattern of dopamine denervation centered on the VTA. One-Way ANOVA showed a significant effect of the groups for the VTA region only [*F*_(3, 43)_ = 57.66; *p* < 0.0001]. In this region, the mean number of TH positive cells was significantly (*p* < 0.05) lower in L and L-PPX groups compared to Sh and Sh-PPX groups (Figure 2A). A decrease of 36% and 33% of TH-positive cell density was observed in L and L-PPX groups respectively compared to sham rats. Lesion of the VTA appeared to be restricted to the medial part of the VTA in L and L-PPX rats (Figure 2B), a region known to mainly project to the accumbens nucleus. On the contrary, the decrease in the SN remained very low (less than 2%) and no significant differences were observed between groups.

### Behavioral results

#### Effect of the daily PPX treatment

**Figure 3:**
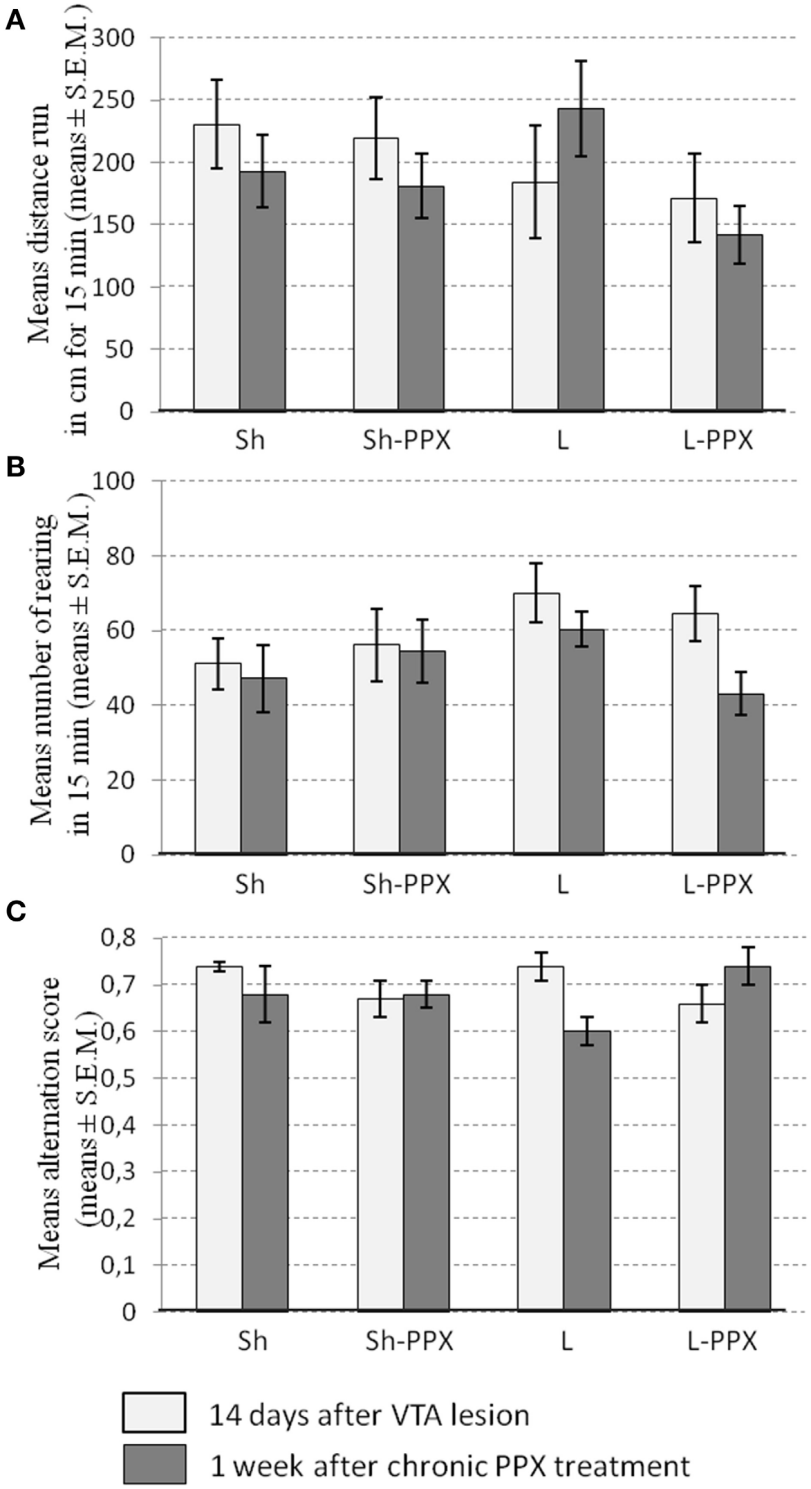
**Daily administration of pramipexole (PPX) has no effect on spontaneous locomotors activity and spontaneous alternation Y maze**. Spontaneous locomotor activity **(A)** and rearing **(B)** were not affected by the VTA lesion (white bars) or by 1 week of daily administration of PPX (gray bars) whatever the experimental group considered. The alternation score measure in Y maze **(C)** was not modified by neither the lesion (white bars) nor 1 week of daily PPX administration (gray bars). Sh, Sham animals; Sh-PPX, sham animals with daily pramipexole treatment (0.1 mg/kg/day); L, VTA lesioned rats; L-PPX, VTA lesioned rats with daily pramipexole treatment (0.1 mg/kg/day).

***Spontaneous locomotor activity in the open field***. The Two-Way ANOVA with repeated measures did not show a significant effect of factors on the mean distance run in the open field arena (Figure 3A) or the amount of rearing (Figure 3B). Overall, the PPX daily administration had no impact on the locomotor activity of rats, whether they were lesioned or not.

***Spontaneous alternation in the Y maze***. The Two-Way ANOVA with repeated measures indicated significant interaction between repetition and treatment [*F*_(1, 43)_ = 8.34; *p* < 0.05] without significant difference in the *post-hoc* tests. We noted that the alternation score in Sh and L rats appeared to decrease slightly 1 week after daily PPX treatment (Figure 3C), whereas its increased slightly in L-PPX rats, although these differences were not significant.

#### Intermittent daily feeding

**Figure 4:**
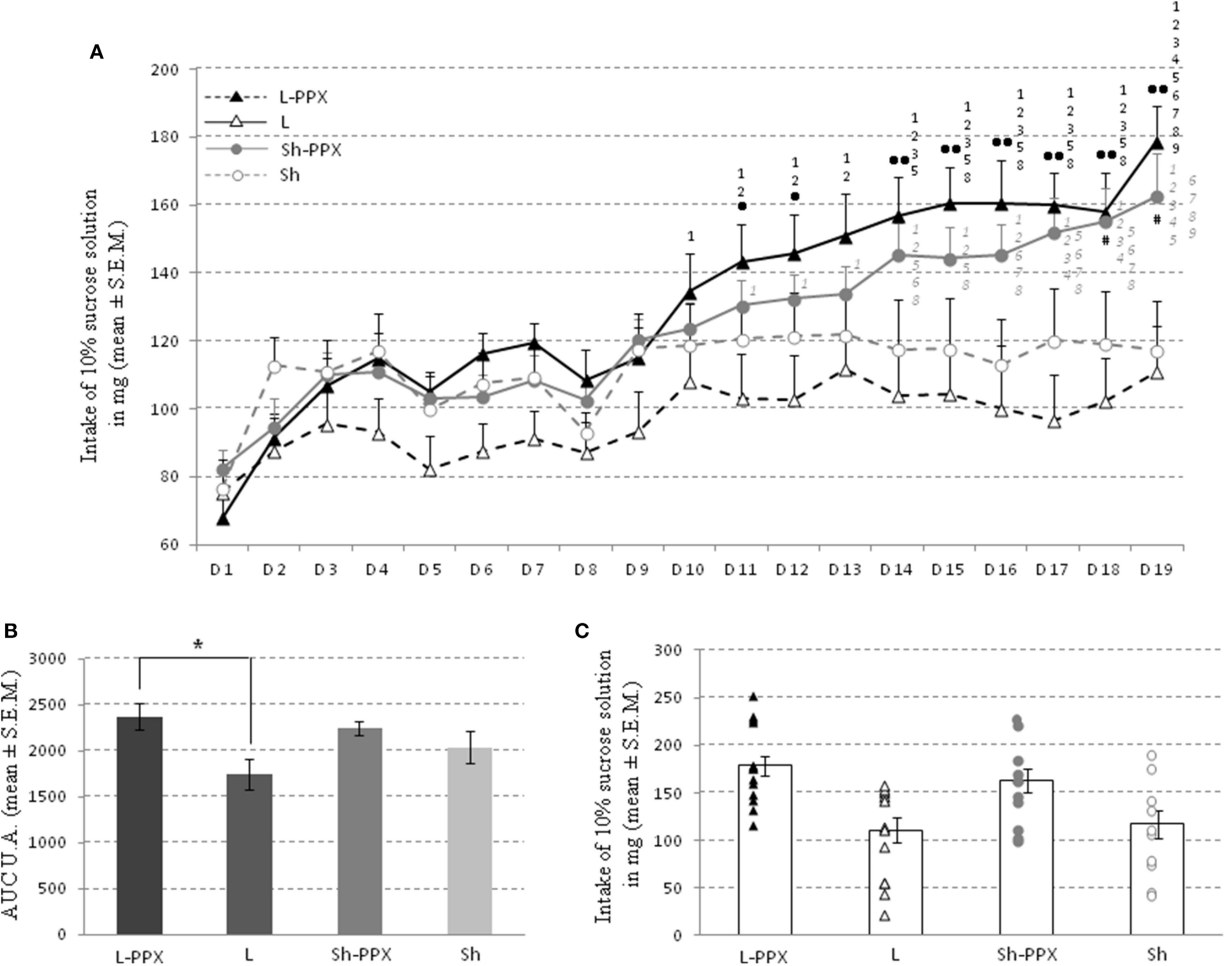
**Sucrose solution consumption during intermittent daily feeding**. Intake of the 10% sucrose solution escalates over the 19 days **(A)** whatever the group considered. Since the consumption of L rats (white triangle and black dash line) and Sh rats (white circles and gray dash line) escalated slowly and stabilized, the intake of Sh-PPX (gray circles and gray bold line) and L-PPX (black triangle and black bold line) rats increased quickly over days. The • represent significant (*p* < 0.05) and •• significant (*p* < 0.001) difference between L and L-PPX rats, the # indicated that Sh-PPx rats were significant (*p* < 0.05) different from L ones. The black number indicates significant (*p* < 0.05) difference with previous days for L-PPX rats, the gray one is for Sh-PPX group. For example the sucrose intake of L-PPX rats was significantly (*p* < 0.05) higher in day 14 compared to day 1, 2, 3, and 5. The area under curves (A.U.C.) analysis **(B)** showed that L-PPX group was significantly (^*^ p<0.05) higher compared to L rats. The intake of 10% sucrose solution of each animals and the mean of each group was represented to observe the potential distribution of rats **(C)**. L-PPX rats: black triangles, L Rats: white triangles, Sh-PPX rats: gray circles and Sh rats: white circles. Sh: Sham animals, Sh-PPX: sham animals with daily pramipexole treatment (0.1 mg/kg/day), L: VTA lesioned rats, L-PPX: VTA lesioned rats with daily pramipexole treatment (0.1 mg/kg/day).

The daily intake of a 10% sucrose solution for the 12 h of the dark period escalated over the days, irrespective of the experimental group considered (Figure 4A). The Two-Way ANOVA for repeated measures indicated a significant effect of treatment [*F*_(1, 43)_ = 8.56; *p* < 0.05], a significant effect of repetition [*F*_(18, 774)_ = 26.8; *p* < 0.0001] and an interaction between repetition and treatment [*F*_(18, 774)_ = 7.48; *p* < 0.0001]. An additional analysis using a One-Way ANOVA with day as factor for each group indicated an effect only in L-PPX and Sh-PPX [respectively *F*_(18, 246)_ = 9.15; *p* < 0.0001 and *F*_(18, 208)_ = 7.91; *p* < 0.0001].

The day-to-day sucrose intake slowly escalated from the first to the tenth day for all experimental groups. In the L group, although rats drank less sucrose solution than the other groups (no significant difference), their intake escalated slowly. On the 14th day, a clear distinction appeared between groups. Since the sucrose solution intake remained stable for the rats receiving saline injections, those treated with pramipexole continued to increase their sucrose intake slowly. The sucrose consumption of rats treated with pramipexole was significantly (*p* < 0.05) higher compared to the saline treated groups on the 18th and 19th days, indicated by the Tukey *post-hoc* test.

Interestingly, in the L-PPX and Sh-PPX groups, the shapes of the sucrose intake escalation curves were similar with a continuous increase of sweet solution intake. From the 11th day to the 13th day, the sweet solution intake for the L-PPX group continued to increase, while it remained mostly constant for the other groups. On day 14 to day 19, the volume of 10% solution consumed by L-PPX rats was significantly (*p* < 0.05) higher compared to L rats. For the Sh-PPX group, sucrose solution intake was significantly (*p* < 0.05) higher compared to L rats, but was never significantly different from Sh rats. In addition, the A.U.C. analysis showed a significant group effect [*F*_(3, 43)_ = 2.80; *p* = 0.05] in a One-Way ANOVA with group. *Post-hoc* tests indicated that the A.U.C. was significantly (*p* = 0.05) higher for the L-PPX group compared to the L (Figures 4B,C).

#### Change in anxiety after removing sucrose access

**Figure 5:**
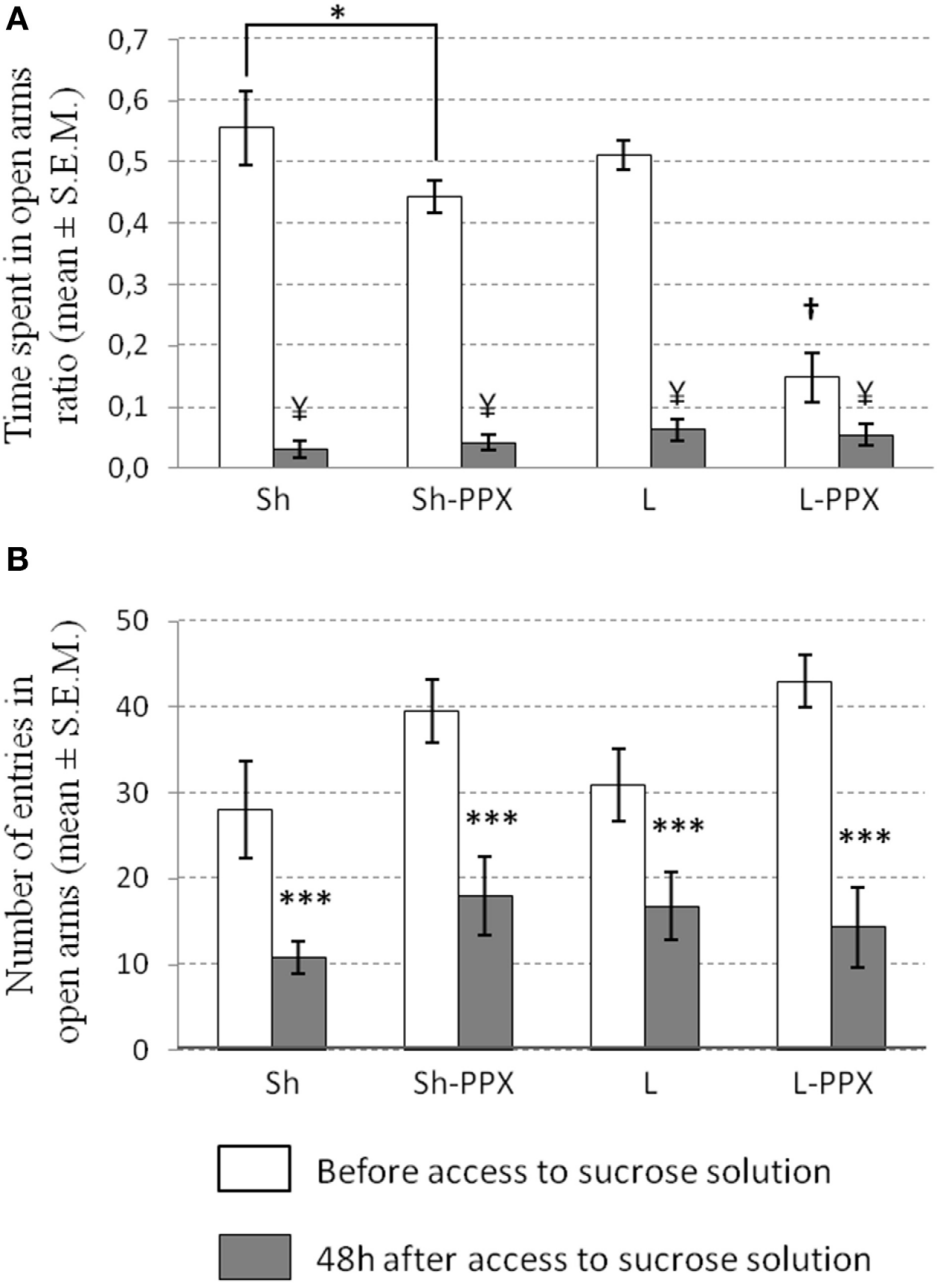
**Effect of sucrose access removal on anxiety measured by elevated plus maze. (A)** Time spent in open arm ratio (OTR) for each experimental groups before intermittent daily access (white bars) and after 2 days of removing access to sucrose solution (gray bars). OTR was significantly lower in L-PPX compared to all other groups before access to sucrose (^†^*p* < 0.001). OTR was also significantly lower in Sh-PPX compared to Sh one (^*^p < 0.05). When access to sucrose was removed to animals, we noted a significant decrease of the OTR in all experimental groups compared to value before access to sucrose solution (¥ *p* < 0.05). **(B)** The number of entries in open arms was significantly decreased (^***^p < 0.0001) when compared before having access to 10% sucrose solution period (white bars) to after removal access period (gray bars). Sh, Sham animals; Sh-PPX, sham animals with daily pramipexole treatment (0.1 mg/kg/day); L, VTA lesioned rats; L-PPX, VTA lesioned rats with daily pramipexole treatment (0.1 mg/kg/day).

***Change in OTR***. The Two-Way ANOVA for repeated measures indicated an effect of repetition [*F*_(1, 43)_ = 28.83; *p* < 0.0001], of treatment [*F*_(1, 43)_ = 17.43; *p* < 0.0001] and a significant interaction between these two factors [*F*_(3, 43)_ = 20.03; *p* < 0.0001] on the OTR ratio measure before and after access to sucrose. Before the initiation of intermittent access to the sucrose solution, the OTR ratio was significantly (*p* < 0.001) lower in the L-PPX groups compared to all the other experimental groups (Figure 5A). In addition, we also noted that this ratio in Sh-PPX rats was significantly (*p* < 0.05) lower compared to that of Sh rats. When the OTR was evaluated again 2 days after removing access to the sucrose solution, we noted a significant (*p* < 0.05) decrease in this ratio in all the animal groups compared to the value before the period of access to sucrose. Nevertheless, we did not observe significant differences in this ratio between the experimental groups when evaluated after the withdrawal of access to sucrose.

***Number of entries in the open arms***. The frequency of entry into the open arms was also analyzed as an index of anxiety. The Two-Way ANOVA for repeated measures indicated a significant effect of the treatment factor [*F*_(1, 43)_ = 4.28; *p* < 0.05] and of repetition [*F*_(1, 43)_ = 70.64; *p* < 0.0001]. Tukey–Kramer *post-hoc* test indicated that saline treated animals were significantly (*p* < 0.05) different from the PPX treated ones (Figure 5B). The decrease of the frequency of entrance between before and after sucrose access was around 58% in Sh animals, 55% in Sh-PPX animals, 50% in L animals, and 64% in L-PPX animals, but no significant differences were observed between groups.

### Change of dopamine D3 receptor expression in the accumbens nucleus region

**Figure 6:**
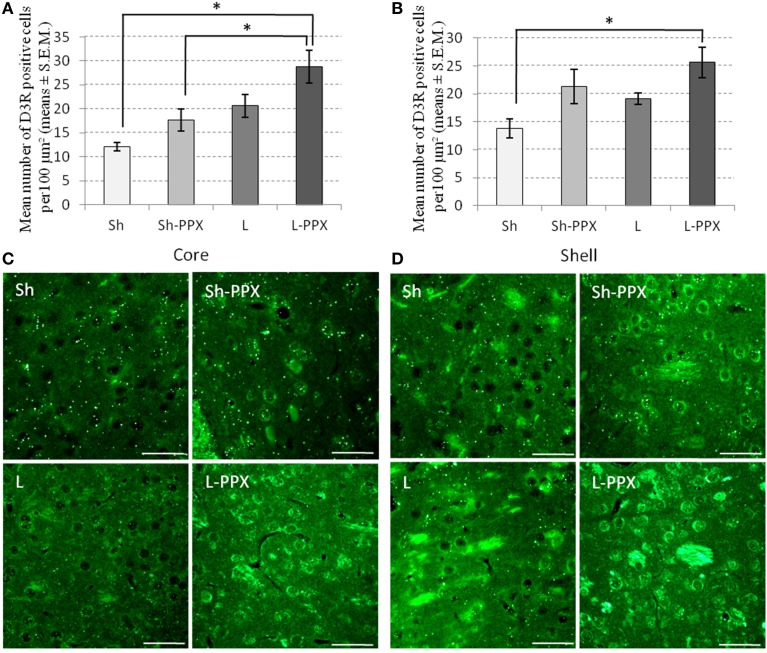
**Changes in Dopamine D3 receptor expression following PPX treatment and intermittent access to sucrose solution**. In the core region of the accumbens nucleus **(A,C)**, the number of D3 positive cells was significantly (^*^p < 0.05) higher in L-PPX rats compared to Sh and Sh-PPX ones. Number of D3 positive cells was also significantly (^*^p < 0.05) higher in the shell region of the accumbens nucleus **(B,D)** of the L-PPX rats compared to Sh ones. Microphotographes are high magnification (×40) of the core **(C)** or the shell **(D)** regions taken at around 2 mm from Bregma. White scales bars represent 200 μm. Sh, Sham animals; Sh-PPX, sham animals with daily pramipexole treatment (0.1 mg/kg/day); L, VTA lesioned rats; L-PPX, VTA lesioned rats with daily pramipexole treatment (0.1 mg/kg/day).

Analysis of D3R immunoreactivity in the core (Figures 6A,C) and shell (Figures 6B,D) regions of the Acb by a One-Way ANOVA indicated a significant effect of the experimental group for these two brain regions [core: *F*_(3, 24)_ = 8.17; *p* < 0.05; shell: *F*_(3, 24)_ = 4.77; *p* < 0.05]. *Post-hoc* tests indicated that the number of positive cells in the core region was significantly (*p* < 0.05) higher in the L-PPX group compared to the Sh and Sh-PPX groups. In addition, in the shell region, the D3R positive cell population was significantly (*p* < 0.05) higher in the L-PPX rats compared to the Sh group.

## Discussion

In this study, we investigated the respective roles of lesion of the VTA and chronic treatment with PPX (32 days) on intermittent daily access to sucrose solution in rats. The main findings are (i) VTA lesion does not change the amount of sweet solution consumed; (ii) PPX chronic treatment induced in sham and lesioned animals a strong increase of tolerance to sucrose solution, sustained by a strong increase of the amount of sucrose solution drank; (iii) an increase of the dopamine 3 receptor in the shell and the core regions of the nucleus accumbens in animals receiving PPX, with a higher expression in lesioned rats compared to sham; (iv) and finally an increase of anxiety behavior in each group of animals after withdrawal of sucrose access.

When intermittent access to the sweet solution was given to sham animals with saline treatment, we observed an escalation of intake followed by a stable phase. This result is in agreement with previous studies of Avena et al. (2008a,b) showing that animals developed a tolerance for sucrose solution intake when given by intermittent access, followed by a stable phase. Thus, intermittent daily feeding has been described as a model of binge eating in animals since it shares common features with substance dependence such as tolerance and signs of withdrawal (Avena, 2010).

Lesion of the VTA did not change the time-course of sucrose intake. This result is in agreement with studies showing that interference with nucleus accumbens dopamine transmission by depletions or antagonisms has little or no effect on food intake (Salamone and Correa, 2012, 2013) and that 6-OHDA lesion of the VTA has no effect on consumption of sucrose in the 2 h test period with a two bottle test (Shimura et al., 2002). Indeed, although it is known that modification of dopaminergic innervations from midbrain VTA to forebrain structures, including the nucleus accumbens in the ventral striatum, decreases effort-related behavior for food, the acquisition of visual-to-sucrose Pavlovian associative learning and development of instrumental goal-directed behavior (Parkinson et al., 2002; Nicola et al., 2005), such manipulation of dopamine leaves the motivation to approach and consume food intact (Salamone and Correa, 2009). Such preservation of motivation could account for the continued sucrose intake escalation observed in lesioned rats. In the context of the intermittent daily feeding experiment, animals did not need to make an association between cues (odor or taste) and sucrose delivery, so they did not need dopamine to trigger associative processes such as “incentive salience attribution” (Berridge and Robinson, 1998). Accordingly, in intermittent daily feeding, it can be assumed that dopamine from the VTA may act only on the initial phase of approach and motivation toward the sucrose solution, while continued escalation could imply other neurotransmitters such as opioids or acetylcholine (Avena et al., 2008a,b) and the action of other brain regions such as the amygdala or prefrontal cortex (Diaz et al., 1995; Hitchcott et al., 1997). An alternative hypothesis is that moderate lesion of VTA, as performed in the present study, leaves compensatory mechanisms intact enough to elicit motivation toward the sucrose solution.

The present data are the first to have been obtained from investigation into the effect of chronic treatment with PPX on day-to-day sucrose intake in animals with lesion of dopaminergic brain system. In both lesioned and sham rats, chronic PPX induced a strong increase of the amount of sucrose solution drank and its ongoing escalation. This result showed that PPX elicited a marked shift in animal tolerance for sucrose. The present results were in agreement with the ones from the study of Willner et al. (1994). They showed that chronic PPX treatment restored 1% sucrose solution intake in mild stressed rats as well as non-stressed rats. In the case of stressed animals, chronic PPX reversed the stress induced anhedonia, but also induced increase of sucrose intake in normal rats. The role of dopamine in food eating behavior remains controversial in humans and animals, there is strong evidence to suggest that dopamine may participate in food seeking. Indeed, the Dopamine D3 receptor agonist participates in reinstating food seeking at larger doses and potentiates food primed response at low doses (Duarte et al., 2003). In humans, PPX induces weight gain and also restores hunger in PD patients (Kumuru et al., 2006). Furthermore, PD patients under PPX can develop compulsive food eating traits as well as gain weight. Interestingly, when PPX is lowered or discontinued, these symptoms disappear or are reduced (Nirenberg and Waters, 2006). Finally, it has been shown that in overweight and obese subjects, Dopamine D3 receptor antagonists decrease attentional bias toward food cues in low-restrained eaters (Nathan et al., 2012). Thus, the main result of the present work indicate that PPX acts on both lesioned and sham animals by inducing higher tolerance for sweet solutions, pointing to a potential role of D3 receptor stimulation in certain aspects of food-related addiction and compulsion. Such a shift in sucrose intake also seemed to be sustained by molecular modifications in the Acb in the present study.

We showed that D3 receptor expression was higher in animals under chronic treatment compared to those under saline treatment. In addition, we also noted that the number of cells expressing the D3 receptor was significantly higher in the core of the Acb of L-PPX rats compared to Sh-PPX ones. The D3 receptor subtype is mainly expressed in the mesocorticolimbic dopamine pathway (Sokoloff et al., 2001). Recent studies have pointed out that the D3 receptor, which is mostly expressed in the Acb, could play an important role in reinforcement and reward (Heidbreder et al., 2005; Chen et al., 2006). Due to its particular pattern of expression, the D3 receptor has drawn attention due to its potential role in the dysregulation of motivated behaviors in humans (Gyertyán and Gál, 2003; Everitt et al., 2008; Besson et al., 2010) and animals (Engelm et al., 2012). It has been proposed that food anticipation, consumption, and unconditioned consummatory behavior (i.e., eating) activate DA release in their terminal fields, especially in the Acb and prefrontal cortex (Phillips et al., 1991; Bassareo and Di Chiara, 1997). Such release also seems to be dependent on the reinforcing attraction of available food and on the motivational state of the animal (Wilson et al., 1995; Martel and Fantino, 1996). One possible explanation for our results could be that chronic PPX administration might have amplified the signal associated with the primary reinforcing effect of sucrose presentation in the Acb which contains the D3 receptor at high density, facilitating food-seeking responses. Indeed, chronic PPX administration is known to increase Dopamine D2 and D3 receptor mRNA, D3 dopamine receptor protein and D3 dopamine receptor binding in the striatum and Acb of animals (Maj et al., 2000; Tokunaga et al., 2012).

In this context, the administration of the D3 preferring agonist before the presentation of the sucrose solution, in addition to the dopamine release elicited by the consumption of palatable food (Bassareo and Di Chiara, 1999), could have interfered with the consummatory aspect of food presentation. Such hyper-activation of the dopaminergic system could have positively modulated approach behavior (such as “searching,” “wanting”) toward the sucrose solution in rats under chronic PPX treatment. However, besides the difference in the pattern of Dopamine D3 receptor positive cells between Sh-PPX and L-PPX, no difference in sucrose tolerance was observed. One possible explanation could be that increased Dopamine D3 receptor expression is only a part of a larger change in brain neurochemistry sustaining tolerance for sucrose. It is known that the lesion of the dopaminergic brain region leads to a profound change in brain neurochemistry and particularly in GABA and glutamate release (Kickler et al., 2009; Chassain et al., 2010, 2013). Such modifications were not explored in the present work and could be addressed in further experiments.

We also noted changes in the expression of anxiety when access to a sweet solution is removed, whatever the experimental groups considered. Such an increase of anxiety in animals after removing access to sucrose has been observed by Hoebel et al. (2007) and Avena et al. (2008b), pointing to opiate-like withdrawal behaviors when sucrose is no longer presented. The change in anxiety may reflect the effect of withdrawal from preferred fluids or drugs that induce negative or aversive states that potentially lead to a relapse of drug consumption (Erb, 2010; Pickens et al., 2011). However, we observed specific changes in anxiety in animals under chronic PPX treatment. L-PPX and, to a lesser extent, Sh-PPX showed increased anxiety compared to their respective controls (L and Sh groups). Such a change in anxiety seems to be linked to dopamine agonist treatment, since the lesion of dopamine system did not modify anxiety in the animal model on its own (Carvalho et al., 2013). The present increase of anxiety in the rats treated could accounts for the higher tolerance for sucrose. Indeed, although the animals were more anxious, they could have been more sensitive to the withdrawal effect observed in the 12 h period when sucrose was not accessible. Anxiety is one of the factors that contributed to addiction or relapse in humans and rats (Erb, 2010).

Our behavioral results also revealed that neither 1 week of daily treatment by PPX, nor the VTA lesion had an impact on spontaneous locomotor activity and spontaneous alternation in the Y maze. This result is in agreement with studies showing that lesion of the VTA has no impact on locomotor activity (Pioli et al., 2008; Drui et al., 2013; Ouachikh et al., 2013). The absence of modification of locomotor activity could be also due to the PPX dose chosen in the present experiment. We used a dose of 0.1 mg/kg based on the study of Collins et al. (2005), showing that such a low dose induced preferential activation of the D3 receptor. In addition, this low dose induced brief hypolocomotion after injection (30 min periods) without inducing hyperlocomotion (Chang et al., 2011). In the present work, in order to avoid a confounding effect of PPX on locomotion, we subjected animals to behavioral tasks at least 2 h after PPX injection. The fact that the animals did not display disturbed locomotion with lesions or with PPX treatment allowed us to validate our results for the other behavioral tasks.

To conclude, the present results showed that long-term stimulation of the D3 receptor using PPX induced an increase in sucrose solution intake as well as in the rate of escalation in sham animals and lesioned rats, along with an increase of the expression of the D3 receptor in the ACb. Despite an increase of D3 receptor expression in Acb compared to sham PPX, no change of sucrose solution intake was observed in lesioned animals receiving PPX, thus showing the main role played by PPX in this behavior compared to the VTA lesion itself.

### Conflict of interest statement

Dr. Franck Durif receives remuneration as a consultant for Novartis, Teva, Lundback, UCB, Aguettant, and Allergan. The authors declare that the research was conducted in the absence of any commercial or financial relationships that could be construed as a potential conflict of interest.
